# Molecular profiling of thalassemia in Southeastern Romania

**DOI:** 10.1007/s12308-026-00732-3

**Published:** 2026-09-09

**Authors:** Costel Stelian Brînzan, Miruna-Gabriela Vizireanu, Anca Florentina Mitroi, Georgeta Camelia Cozaru, Mariana Așchie, Florina Madalina Oniceanu, Adrian Nelutu Mitroi, Ionut Eduard Iordache

**Affiliations:** 1 Sf. Apostol Andrei Clinical Emergency County Hospital, 145 Tomis Blvd, Constanta, Romania; 2 Faculty of Medicine, CEDMOG Center, Ovidius University, 145 Tomis Blvd, Constanta, Romania; 3 Medical Sciences Section, Academy Of Romanian Scientists, București, Romania; 4 CFR Hospital, Constanta, Romania

**Keywords:** α-Thalassemia, β-Thalassemia, δβ-Thalassemia, Southeastern, Romania

## Abstract

**Background:**

Thalassemias represent a major group of hereditary hemoglobin disorders with significant global health impact, yet molecular data from Eastern European populations, particularly from Romania, remain limited.

**Purpose:**

This study was aimed at comprehensively characterizing the molecular spectrum of α-, β-, and δβ-thalassemia in the southeastern part of Romania using an expanded panel of molecular diagnostic techniques.

**Methods:**

A total of 324 patients with suspected thalassemia were evaluated between 2017 and 2025. Molecular testing included multiplex PCR with reverse dot-blot hybridization, Sanger sequencing, MLPA for α-globin deletions/duplications, and multiplex gap-PCR for δβ fusion gene detection.

**Results:**

Pathogenic variants were identified in 137 individuals (42.28%). Of these, 21.89% had α-thalassemia, 66.42% had β-thalassemia, 10.21% had combined α and β defects, and 1.45% had Lepore Hb variants. The −α^3^·^7^ deletion and anti-α^3^·^7^ triplication were the predominant α-globin abnormalities, while IVS I-6 [T > C], IVS I-110 [G > A], codon 39 [C > T], and IVS II-745 [C > G] were the most frequent β-globin mutations. Two cases of the Hb Lepore Boston-Washington subtype were confirmed through gap-PCR and sequencing.

**Conclusions:**

The mutation distribution observed in southeastern Romania mirrors Mediterranean thalassemia patterns rather than those of Central or Northern Europe.

**Supplementary Information:**

The online version contains supplementary material available at https://doi.org/10.1007/s12308-026-00732-3.

## Introduction

Thalassemia constitutes a broad spectrum of inherited disorders of hemoglobin production, transmitted in an autosomal recessive pattern, which arise from imbalances in the synthesis of α- and/or β-globin chains. The inadequate production of these polypeptides disrupts hemoglobin assembly and gives rise to hypochromic microcytic anemia. The conditions associated with α- and β-globin deficiencies most commonly arise from structural alterations, deletions, duplication, point mutations, or defects affecting regulatory regions within the *HBA1/HBA2* loci on chromosome 16 and the *HBB* gene on chromosome 11 [1, 2].

Clinically, thalassemia is classified into mild (carrier state), intermediate, and severe forms according to the degree of anemia [3]. Patients with mild thalassemia typically exhibit slight hypochromic microcytic anemia [4], whereas those with intermediate forms often present with more pronounced symptoms and may require intermittent blood transfusions [5]. Severe thalassemia is associated with major clinical complications, including hydrops fetalis in α-thalassemia [6] and neonatal jaundice, craniofacial deformities (such as prognathism), and hepatosplenomegaly in β-thalassemia [7]. Without appropriate treatment, children with β-thalassemia major frequently die before the age of five [8]. Patients with severe forms require lifelong, regular blood transfusions combined with iron-chelation therapy [9], imposing a significant socioeconomic burden on affected families and healthcare systems [10].

Worldwide, approximately 5% of the population carries a hemoglobinopathy-associated variant, with an estimated rate of 5% individuals harboring at least one α-thalassemia mutation; in comparison, approximately 1.5% (80–90 million people) are carriers of β-thalassemia [11]. Thalassemia is highly prevalent in tropical and subtropical regions, including the Mediterranean basin, the Middle East, North Africa, India, and Southeast Asia (SEA) [12]. Due to increased global migration, however, thalassemia traits and related disorders are now encountered far beyond their traditional endemic regions [13]. In Romania, β-thalassemia trait appears to be relatively uncommon, with an estimated prevalence of approximately 0.5% in the general population, and a cumulative incidence of ~ 0.32% reported in a pediatric cohort from Constanța County [14]. Robust nationwide epidemiological data on α-thalassemia prevalence remains limited [15].

Large deletions involving the *HBA1* and *HBA2* genes account for approximately 85–90% of all α-thalassemia cases [16]. The most frequent pathogenic variant is the −α^3^·^7^ deletion, the most common α-globin deletion worldwide, followed by the −α^4^·^2^ deletion, the 20.5-kb deletion, and large double-gene deletions such as the South-East Asian (–SEA) and Filipino (–FIL) deletions [6]. In addition to these, clinically relevant non-deletional variants include single-nucleotide substitutions, most notably *HBA2*:c.427T > C (Hb Constant Spring), *HBA2*:c.377T > C (Hb Quong Sze), and *HBA2*:c.369C > G (Hb Westmead) [17].

In contrast, β-thalassemia results primarily from over 200 known pathogenic point mutations in the *HBB* gene, predominantly single-nucleotide variants and small insertions or deletions (indels) affecting exons, intronic splice sites, or promoter regions [18]. The sickle-cell mutation (*HBB*:c.20A > T, HbS) is the most prevalent pathogenic variant of *HBB* worldwide [19]. Although several *HBB* mutations have a broad global distribution, many exhibit strong geographic specificity, particularly among populations from China and Southeast Asia, the Middle East, and the Mediterranean region [12].

Hb Lepore represents a heterogeneous group of thalassemia-like hemoglobinopathies characterized by deletions within the β-globin gene cluster, which result in the fusion of portions of the δ- and β-globin genes. This rearrangement leads to the production of a δβ-hybrid globin chain, composed of the N-terminal region of the δ-globin and the C-terminal region of the β-globin chain. Three major structural variants of Hb Lepore have been identified, each defined by distinct breakpoint loci. Hb Lepore Boston-Washington results from a crossover between δ codon 87 and either β intron 2 nucleotide 8 or β codon 116. In contrast, Hb Lepore Hollandia arises from a breakpoint located between δ codon 22 and β codon 50. Hb Lepore Baltimore is associated with deletions involving regions between δ codon 50 and β codon 86, δ codon 68 and β codon 84, or δ codon 59 and β codon 86 [20].

This study was aimed at characterizing the molecular spectrum of α-thalassemia, β-thalassemia, and δβ-thalassemia/Lepore variants in southeastern Romania, including patients in whom hemoglobin electrophoresis results were suggestive of thalassemic syndromes or structural hemoglobin defects. To achieve this, a molecular diagnostic approach was applied. The genotyping strategy included reverse dot-blot hybridization, Sanger sequencing for the detection of SNVs and small indels, as well as gap-PCR and multiplex ligation-dependent probe amplification (MLPA) for the identification of gene deletions and other large rearrangements.

## Material and methods

Between January 2017 and November 2025, a total of 324 patients with suspected thalassemia were referred for molecular testing to the Medical Genetics Laboratory, part of the Clinical Pathology Department at the Emergency County Clinical Hospital Constanța, Romania. Patients were referred by hematologists based on clinical suspicion of thalassemia, following routine complete blood count (CBC) and hemoglobin electrophoresis findings suggestive of a thalassemic syndrome. The cohort comprised 167 males and 157 females originating from the Departments of Pediatrics and Hematology-Oncology. The inclusion criteria were microcytosis (MCV < 80fL) and hypochromia (MCH < 27 pg) identified on routine blood testing, and/or a positive family history of thalassemia (particularly from regions with specific ethnic backgrounds). The patients’ ages ranged from 1 to 71 years. Demographic and clinical information, including sex, age, and relevant laboratory data, was retrieved from the Electronic Medical Record System. This study was designed to characterize the molecular spectrum of α-, β-, and δβ-thalassemia variants in patients who had already undergone clinical and hematological pre-screening by the referring hematologists.

This study was approved by the Local Ethics Commission for Clinical and Research Developmental Studies of the Emergency County Clinical Hospital Constanța, and written informed consent was obtained from all participants before sample collection. Two milliliters of peripheral venous blood were collected from each patient into EDTA tubes. Genomic DNA was extracted from 200 μL of whole blood using the QIAamp Blood Mini Kit (Qiagen, Hilden, Germany), following the manufacturer’s instructions. Molecular testing for the α- and β-globin gene regions was performed using multiplex PCR followed by inverse dot-blot hybridization, which employed biotin-labeled primers targeting these regions. The resulting amplification products were detected through hybridization to allele-specific oligonucleotide probes immobilized as parallel lines on the assay strips, in accordance with the commercial kits: the α-Globin StripAssay® (ViennaLab Diagnostics, Vienna, Austria), which detects 21 common α-thalassemia mutations, and the β-Globin StripAssay® MED (ViennaLab Diagnostics, Vienna, Austria), which screens for 22 common β-thalassemia mutations.

For the confirmation of deletions/duplications and associated regulatory regions within the α-globin gene cluster, the SALSA MLPA Probemix P140-B4 *HBA* (MRC-Holland, Amsterdam, The Netherlands) assay was performed according to the manufacturer’s instructions. Briefly, 5 µL of genomic DNA (10 ng/µL) was denatured at 98 °C for 5 min and cooled to 25 °C. Subsequently, 1.5 µL of the probe mix and 1.5 µL of SALSA hybridization buffer were added, followed by denaturation at 95 °C for 1 min and hybridization at 60 °C for 18 h. Ligation was carried out by adding 32 µL of Ligase-65 master mix (3 µL Ligase Buffer A, 3 µL Ligase Buffer B, 1 µL Ligase-65 enzyme, and 25 µL RNase/DNase-free water) to each sample, with incubation at 54 °C for 15 min, followed by enzyme inactivation at 95 °C for 5 min and cooling to 20 °C. PCR amplification was then performed using 10 µL of PCR master mix (comprising 0.5 µL SALSA polymerase, 2 µL SALSA primer mix, and 7.5 µL RNase/DNase-free water), added to each tube containing the ligated product. PCR cycling conditions were as follows: 95 °C for 30 s, 60 °C for 30 s, and 72 °C for 60 s, repeated for 35 cycles. This was followed by a final extension at 72 °C for 20 min and a hold at 15 °C. For fragment sizing, 0.2 µL of the GeneScan™ 500 LIZ® (Applied Biosystems, Foster City, CA, USA) size standard was added to each reaction. PCR products were analyzed by capillary electrophoresis using an ABI 3500 Genetic Analyzer, and data normalization and interpretation were performed with Coffalyser software (v. 250317.1029, MRC-Holland, Amsterdam, The Netherlands).

The δβ fusion gene was identified through multiplex gap-PCR using a universal forward primer and variant-specific reverse primers. The Gap-PCR was performed on a Biometra T-Advanced thermal cycler (Analytik Jena, Jena, Germany) using the following protocol: 2 × Platinum II Hot-Start PCR Master Mix (Invitrogen, Thermo Fisher Scientific, Waltham, MA, USA), 0.4 μL of each primer (10 μM), 5 μL of template DNA, and ultrapure water to reach a total volume of 20 μL. The cycling program started with an initial denaturation at 94 °C for 2 min, followed by 35 cycles of 94 °C for 15 s, 60 °C for 15 s, and 68 °C for 30 s, ending with a final hold at 4 °C. Post-PCR, the products were separated by 3% agarose gel electrophoresis, which revealed distinct banding patterns characteristic of each variant: Hb Lepore Boston-Washington (616 bp), Hb Lepore Baltimore (552 bp and 616 bp), and Hb Lepore Hollandia (359 bp, 552 bp, and 616 bp). An internal control (*LIS1* gene) and wild-type Hb Lepore primers generated control fragments of 197 bp and 439 bp, respectively. Precise breakpoint confirmation for all three variants was achieved through capillary electrophoresis on an ABI 3500 Genetic Analyzer, with sequence data analyzed using CodonCode Aligner (version 9.0.1, CodonCode Corporation, Centerville, MA, USA).

### Statistical analysis

Statistical analysis was performed using descriptive methods. Categorical variables were summarized by percentage and frequency and number of cases was reported for each group. Graphical representation of frequency was created using R version 4.5.2 (R Core Team, 2025).

## Results

**Fig. 1:**
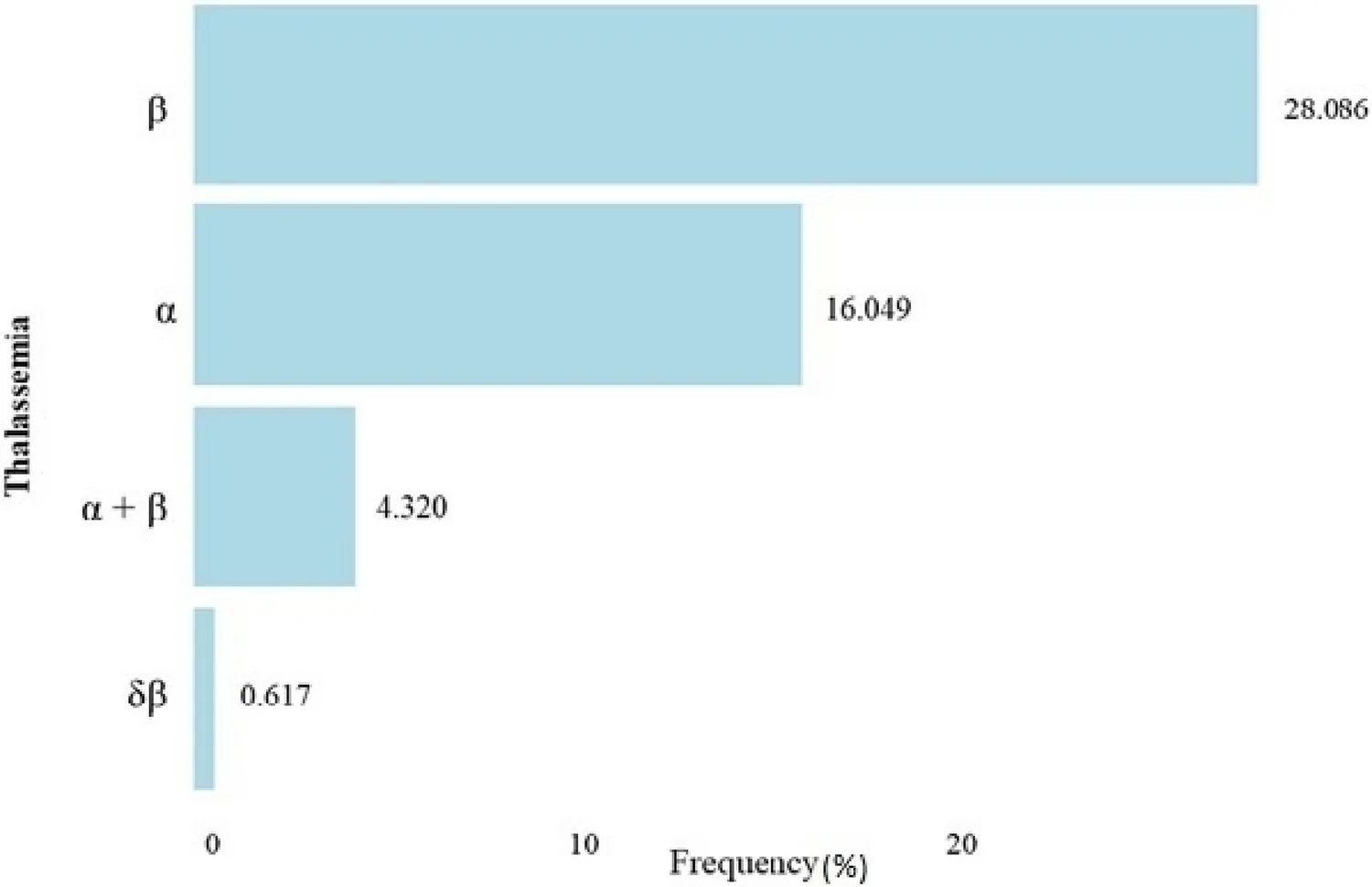
Prevalence of α-thalassemia, β-thalassemia, combined α + β thalassemia, and δβ-thalassemia in our study. Bars represent the percentage frequency of each thalassemia category among all individuals with mutations. β-Thalassemia was the most prevalent form (28.086%), followed by α-thalassemia (16.049%), combined α + β genotypes (4.32%), and δβ-thalassemia (0.617%)

The study enrolled a total of 324 subjects, among whom 137 cases (42.28%) were found to have mutations. These cases included 30 (21.89%) α-thalassemia, 91 (66.42%) β-thalassemia, 14 (10.21%) combined α- and β-thalassemia, and 2 (1.45%) Lepore Hb Variants (Fig. 1).

### Genotypes of α-thalassemia

Among the 30 subjects diagnosed with α-thalassemia mutations within the cohort, a total of 7 distinct α-globin genotypes were identified (Table 1), where −α^3^·^7^/αα single-gene deletion was the most frequent abnormality in the cohort, accounting for 22 cases. One patient was homozygous for the −α^3^·^7^/−α^3^·^7^ genotype.

**Table 1:** Prevalence rate and mutation spectrum of α-thalassemia

Genotypes	Phenotype	Cases	Frequency (%)
−α^3.7^/αα	α^+^/α	22	6.79
−α^3.7^/−α^3.7^	α^+^/α^+^	1	0.30
−α^4.2^/αα	α^+^/α	1	0.30
––20.5/αα	α^0^/α	1	0.30
––MED/αα	α^0^/αα	3	0.92
α2 poly A-1 [AATAAA > AATAAG]/αα	α^+^/αα	1	0.30
−α^3.7^/αα + α2 poly A-2/α2 poly A-2	α^+^/α^+^	1	0.30

Other deletional variants were less common, including the -MED/αα double-gene deletion (3 cases), the −α^4^·^2^/αα deletion (1 case), and the −20.5/αα double-gene deletion (1 case). A rare non-deletional α-thalassemia variant was also identified (1 case), namely, the α2 polyA-1 [AATAAA > AATGAA]/αα signal mutation. In addition, a compound genotype involving −α^3^·^7^/αα and α2 polyA-2 [AATAAA > AATAAG]/α2 polyA-2 was identified in one patient. Deletions and duplications associated with the α-globin gene cluster were confirmed by the MLPA technique (Supplementary figures S1 and S2).

### Genotypes of β-thalassemia

A total of 91 subjects with β-thalassemia were identified, in whom 11 different β-globin genotypes were documented (Table 2). The most common genotype was IVS I-6 [T > C], accounting for 19 cases, comprising 14 in a heterozygous state (IVS I-6 [T > C]/β^N^) and 5 in a homozygous state (IVS I-6 [T > C]/IVS I-6 [T > C]), as well as IVS I-110 [G > A]/β^N^, identified in 19 heterozygous cases, followed by codon 39 [C > T]/β^N^, detected in 15 heterozygous cases. Other prevalent heterozygous mutations included IVS II-745 [C > G]/β^N^ in 12 cases and codon 8 [-AA]/β^N^ in 10 cases. Moderately frequent variants in heterozygous form included IVS I-1 [G > A]/β^N^ and IVS II-1 [G > A]/β^N^, each detected in 5 cases. Several mutations were identified at very low heterozygous frequencies (2 cases each), including codon 5 [-CT]/β^N^ and 87 [C > G]/β^N^. In addition, a compound heterozygous genotype involving IVS I-6 [T > C]/β^N^ and IVS II-745 [C > G]/β^N^ was identified in two patients.

**Table 2:** Prevalence rate and mutation spectrum of β-thalassemia

Genotypes	Phenotype	Cases	Frequency
codon 87 [C > G]/β^N^	β⁺/β^N^	2	0.61
codon 5 [-CT]/β^N^	β^0^/β^N^	2	0.61
codon 8 [-AA]/β^N^	β^0^/β^N^	10	3.08
IVS I-1 [G > A]/β^N^	β^0^/β^N^	5	1.54
IVS I-6 [T > C]/β^N^	β⁺/β^N^	14	4.32
IVS I-6 [T > C]/IVS I-6 [T > C]	β⁺/β⁺	5	1.54
IVS I-110 [G > A]/β^N^	β⁺/β^N^	19	5.86
codon 39 [C > T]/β^N^	β^0^/β^N^	15	4.62
IVS II-1 [G > A]/β^N^	β^0^/β^N^	5	1.54
IVS II-745 [C > G]/β^N^	β⁺/β^N^	12	3.70
IVS I-6 [T > C]/β^N^ + IVS II-745 [C > G]/β^N^	β⁺/β⁺ (compound heterozygous)	2	0.61

### Genotypes of combined* α* + *β-*thalassemia

Fourteen cases of combined α- and β-thalassemia were identified in the cohort (Table 3). A total of 8 distinct combined genotype patterns were observed.

**Table 3:** Prevalence rate and mutation spectrum of combined α + β-thalassemia

Genotypes	Phenotype	Cases	Frequency
ααα/αα; codon 39 [C > T]/β^N^	ααα/αα; β^0^/β^N^	2	0.61
ααα/αα; IVS I-6 [T > C]/β^N^	ααα/αα; β⁺/β^N^	5	1.54
ααα/αα; IVS I-6 [T > C]/IVS I-6 [T > C]	ααα/αα; β⁺/β⁺	2	0.61
ααα/αα; IVS I-6 [T > C]/β^N^ + IVS II-745 [C > G]/β^N^	ααα/αα; β⁺/β⁺ (compound heterozygous)	1	0.30
ααα/αα; IVS II-1 [G > A]/β^N^	ααα/αα; β^0^/β^N^	1	0.30
ααα/αα; IVS II-745 [C > G]/β^N^	ααα/αα; β⁺/β^N^	1	0.30
−α^3.7^/αα, α2 poly A-2/α2 poly A-2; IVS I-6 [T > C]/IVS I-6 [T > C]	α⁺/α⁺; β⁺/β⁺	1	0.30
−α^3.7^/αα; IVS II-745 [C > G]/β^N^	α⁺/α; β⁺/β^N^	1	0.30

The most frequent associations involved anti-α^3^·^7^ triplication in combination with IVS I-6 [T > C] (7 cases) and codon 39 [C > T] (2 cases). Less frequent combinations, each detected in a single case, included *anti-α*^*3*^*·*^*7*^ triplication with IVS II-1 [G > A], IVS II-745 [C > G], and the compound β-genotype IVS I-6 [T > C] + IVS II-745 [C > G]. In addition, one case showed the −α^3^·^7^/αα deletion together with IVS II-745 [C > G], while another presented a complex genotype consisting of −α^3^·^7^/αα + α2 polyA-2 [AATAAA > AATGAA] associated with IVS I-6 [T > C].

Anti-α^3^·^7^ triplication was observed in combination with several β-thalassemia mutations. This configuration is clinically relevant because the increased α-globin gene dosage may exacerbate β-thalassemia severity by increasing the α:β globin chain imbalance. In contrast, the −α^3^·^7^ deletion was identified in only two combined genotypes.

### Lepore Hb variants

In patients with persistently high Hb F levels, the δβ fusion gene was examined in 10 individuals, and a deletion consistent with the Hb Lepore Boston-Washington (δ87/β116) heterozygous subtype was found in 2 of them (Supplementary figure S3).

## Discussion

The molecular landscape of thalassemia in Romania remains an area requiring continuous updates, given the historical and demographic shifts of the region. While thalassemia is globally recognized as a group of common monogenic autosomal recessive disorders that contribute substantially to global morbidity and mortality in Mediterranean, Middle Eastern, and Southeast Asian populations, its distribution in Romania exhibits distinct regional characteristics [21, 22]. The two major forms, α-thalassemia and β-thalassemia, result from pathogenic variants affecting the *HBA1*, *HBA2*, and *HBB* genes, leading to impaired globin chain synthesis and defective hemoglobin production [23]. In the present cohort, the β-thalassemia mutation spectrum closely reflected the typical Mediterranean profile, with point mutations predominating over large deletions. The most frequent variant was IVS I-6 [T > C] *(HBB:*c.92 + 6 T > C*)*, followed by IVS I-110 [G > A] *(HBB:*c.93-21G > A*)* and codon 39 [C > T] *(HBB:*c.118C > T*).* Together, these variants constitute the core Mediterranean β-thalassemia mutation spectrum. Talmaci et al. [24] previously identified IVS I-110 (G > A) and codon 39 (C > T) as the most prevalent β-thalassemia mutations in Romania, whereas IVS I-6 was not among the leading alleles. The predominance of IVS I-6 observed in our cohort may reflect regional genetic heterogeneity within Romania or differences in cohort composition, referral patterns, and sample size. Interestingly, our findings align more closely with those of İnce et al. [25], who reported IVS I-110, IVS I-6, codon 8 (–AA), IVS II-1, and IVS II-745 as the predominant mutations in southeastern Mediterranean populations. Other recurrent variants identified in our study included IVS II-745 [C > G], codon 8 (–AA), IVS I-1 [G > A], IVS II-1 [G > A], codon 5 [–CT], and the promoter variant − 87 [C > G], further illustrating the molecular heterogeneity characteristic of Mediterranean β-thalassemia [24–27]. This contrasts with reports from Northern and Central European countries, where β-thalassemia has historically been uncommon and the current mutation spectrum is increasingly influenced by recent migration from endemic regions rather than by long-standing regional mutation patterns [28]. Overall, these findings suggest that the β-thalassemia mutation spectrum in southeastern Romania is more strongly influenced by Mediterranean genetic backgrounds than by patterns reported in other Romanian regions.

In this study, data on thalassemia were collected from a large group of 324 patients evaluated at the Emergency County Clinical Hospital in the southeastern part of Romania using a molecular diagnostic approach. The distribution of thalassemia genotypes in this population was thoroughly characterized. The overall percentage of individuals carrying pathogenic thalassemia deletions/mutations was 42.28% (137 out of 324). Among these, 21.89% were carriers of α-thalassemia mutations, 66.42% were β-thalassemia carriers, 10.21% carried both α- and β-thalassemia mutations, and 1.45% were Lepore Hbs. The proportion of β-thalassemia carriers identified here 25.9% (84/324), contrasts sharply with the reported β-thalassemia trait prevalence of approximately 0.5% in the general Romanian population [14]. This discrepancy should be interpreted with caution and most likely reflects a substantial referral/sampling bias rather than a true shift in population-level prevalence. Patients in this cohort were not randomly sampled from the general population; rather, they were referred for molecular testing by hematologists following routine CBC and hemoglobin electrophoresis findings suggestive of a thalassemic syndrome. As such, the cohort is enriched by design for individuals with a high prior probability of carrying a thalassemia-associated variant.

The −α^3^·^7^ single-gene deletion identified within the cohort is the most common α-thalassemia variant worldwide, including in Southeast Asia [29]. However, large double-gene deletions, while comparatively rare in the cohort, are considerably more prevalent in parts of Southeast Asia, particularly in northern Thailand and Laos, than in Mediterranean and Middle Eastern populations [15]. The mutation spectrum observed in the study’s cohort, dominated by single-gene deletions and low non-deletional variant frequencies, is therefore more consistent with patterns reported in Mediterranean and Middle Eastern regions than with the elevated double-gene deletion burden seen in specific Southeast Asian populations. The most common deletion was the −α^3^·^7^/αα single-gene deletion, a pattern widely reported in Mediterranean countries, including Italy, Greece, Spain, Cyprus, and Turkey [30]. Other deletional variants, including ––MED/αα, −α^4^·^2^/αα, and ––20.5/αα, were detected at low frequencies, consistent with data from Southern Europe, where the MED and 20.5-kb deletions occur sporadically. A rare non-deletional variant*, α2 polyA-2*, was also identified, a mutation that has been described occasionally in Turkish populations [31]. These patterns reflect the shared genetic and demographic backgrounds of Mediterranean populations, supporting the conclusion that the Romanian mutation spectrum is more closely related to Mediterranean α-thalassemia profiles. Only two homozygous cases were identified in our cohort −α^3^·^7^/−α^3^·^7^ and α2 polyA-1/α2 polyA-2 (Saudi type)—each representing 3.33% of α-thalassemia cases.

A notable finding was the high frequency of anti-α^3^·^7^ triplication, which results from misalignment and unequal crossover between the homologous X-, Y*-*, and Z*-*box regions of the α-globin gene cluster during meiosis. This variant appears to be ubiquitous and has been reported across Mediterranean populations [32]. It carries clinical significance because it can exacerbate the phenotype when co-inherited with β-thalassemia mutations [33].

Fourteen cases of combined α- and β-thalassemia were identified, in which patients carried at least one α-globin mutation together with a β-globin mutation. This finding underscores the importance of comprehensive globin gene testing in populations where both mutation types commonly coexist, as observed throughout the Mediterranean basin [33]. Eight distinct combined genotypes were identified. The most frequent associations involved *anti-α*^*3*^*·*^*7*^ triplication co-inherited with IVS I-6 [T > C] and codon 39 [C > T], genotypes that are well documented in Mediterranean clinical practice. Previous studies by Ropero et al. [34] and Constanço et al. [35] have demonstrated that co-inheritance of anti-α^3^·^7^ triplication can significantly modulate the β-thalassemia phenotype, including cases associated with IVS I-110 and other β-globin variants. Less frequent combinations included anti-α^3^·^7^ triplication with IVS II-1 [G > A], IVS II-745 [C > G], and a compound β-genotype comprising IVS I-6 [T > C] and IVS II-745 [C > G]. Additional patterns involved the −α^3^·^7^/αα deletion in combination with IVS II-745 [C > G], as well as a complex genotype consisting of −α^3^·^7^/αα together with α2 polyA-2 and IVS I-6 [T > C]. Co-inheritance of anti-α^3^·^7^ triplication with β-thalassemia mutations is known to exacerbate the clinical phenotype by increasing the α:β globin chain imbalance.

Hb Lepore consists of two normal α-globin chains paired with two δβ fusion chains generated through unequal crossing-over events between the δ- and β-globin genes. Among the known Hb Lepore variants, Hb Lepore Boston-Washington is the most prevalent and has been reported at low frequencies across several ethnic groups, particularly in Mediterranean populations [20]. Within the analyzed cohort, consistent with previous studies from the Mediterranean region [36–39], screening identified two heterozygous cases corresponding to the Hb Lepore Boston-Washington subtype (δ87/β116) among ten patients who presented with mildly elevated Hb F levels that did not exceed 5%. Clinically, the Hb Lepore Boston-Washington variant typically resembles a β⁺ thalassemia phenotype [38]. Although uncommon, δβ-thalassemia should be considered in the differential diagnosis of individuals with increased Hb F levels and mild microcytic anemia [40].

This study employed a molecular diagnostic approach for the detection of α-thalassemia, β-thalassemia, and δβ-thalassemia. However, some limitations were identified. Socioeconomic constraints may limit availability of molecular assays modifying the genotypic landscape demonstrated here. The molecular methods used in this study were targeted approaches that detect only variants included in the commercial panels. In addition, gap-PCR analysis was restricted to the detection of Hb Lepore rearrangements and did not include screening for other deletions, larger than 619 bp, involving the β-globin gene cluster. Consequently, rare structural β-globin variants and less common or novel SNVs/small indels may have remained undetected. The southeastern region of Romania is characterized by a diverse, multiethnic population, which may introduce genetic heterogeneity and make it challenging to extrapolate these findings to other Romanian regions.

## Conclusion

This study provides a detailed molecular characterization of α-, β-, and δβ-thalassemia in a large cohort from the southeastern part of Romania. The mutation spectrum observed closely aligns with Mediterranean profiles, with a predominance of the −α^3^·^7^ deletion in α-thalassemia, and IVS I-6 [T > C], IVS I-110 [G > A], and codon 39 [C > T] as the most frequent β-globin mutations. Overall, these findings support the implementation of integrated molecular diagnostic strategies to improve thalassemia detection, clinical management, and genetic counseling in Romania.

## Supplementary Information

Below is the link to the electronic supplementary material.

ESM 1 (JPEG 143 KB)

ESM 2 (DOCX 3.05 MB)

## Data Availability

No datasets were generated or analysed during the current study.

## Abbreviations

*HBA*: Hemoglobin alpha
*HBB*: Hemoglobin beta
SEA: Southeast Asia
FIL: Filipino
SNVs: Single nucleotide variants
PCR: Polymerase chain reaction
MLPA: Multiplex ligation-dependent probe amplification
EDTA: Ethylenediaminetetraacetic acid
DNA: Deoxyribonucleic acid
*LIS1*: Lisencephaly-1
MED: Mediterranean deletion
IVS: Intervening sequence
Hb F: Fetal hemoglobin
Bp: Base pair
