# Supplementary material for: Molecular profiling of thalassemia in Southeastern Romania
Source: J Hematop. 2026 Sep 9;19(1):53. doi: 10.1007/s12308-026-00732-3 (PMC13553599; doi:10.1007/s12308-026-00732-3)
Supplement: Supplementary file 1 [file 12308_2026_732_MOESM2_ESM.docx]

**Molecular Profiling of Common α-, β-, and δβ-Thalassemia Mutations in Southeastern Romania**

**Journal of Hematopathology**

Costel Stelian Brînzan, Miruna-Gabriela Vizireanu, Anca Florentina Mitroi*, Georgeta Camelia Cozaru, Mariana Așchie, Florina Madalina Oniceanu, Adrian Nelutu Mitroi and Ionut Eduard Iordache

*Corresponding author: Anca Florentina Mitroi

Sf. Apostol Andrei Clinical Emergency County Hospital, 145 Tomis Blvd, Constanta, Romania;

CEDMOG Center and Faculty of Medicine, Ovidius University, 145 Tomis Blvd., Constanta, Romania;

E-mail: anca.mitroi@365.univ-ovidius.ro

Phone: +40723398798

**Supplementary figure 1** Graph illustrating the *–α³·⁷* single-gene deletion. The x-axis displays the individual MLPA probes spanning the α-globin gene cluster. At the same time, the y-axis represents the ratio calculated as the normalized signal intensity of the proband relative to the reference samples. Probes mapping to the deleted *–α³·⁷* region show reduced ratios of approximately ~0.5 (highlighted by red dots), consistent with the loss of one α-globin gene copy (heterozygous deletion). Ratios around 1.0 indicate normal copy number, while values approaching 0 would indicate biallelic loss. The remaining probes, including those outside the deleted region and the reference probes (grey panel), display ratios within the expected normal range, confirming a single-gene (*–α³·⁷/αα*) deletion.

**Supplementary figure 2** Graph showing the presence of an *anti-α³·⁷* pattern. The x-axis displays the individual MLPA probes across the α-globin gene cluster. At the same time, the y-axis represents the dosage ratio calculated as the normalized signal intensity of the proband relative to the reference samples. Probes within the *HBA2*–*HBA1* region show elevated ratios ranging from ~1.4 to ~1.8 (blue dots), consistent with a gain of one additional α-globin gene copy (α-globin triplication). Ratios around 1.0 indicate normal copy number, while values of ~0.5 and 0 signify loss of one or both alleles, respectively. The elevated probe intensities above the upper reference boundary (blue line) confirm the presence of the *anti-α³·⁷* triplication.

**Supplementary figure 3** Multiplex gap-PCR for the detection of Hb Lepore variants in a 3% agarose gel. Expected amplicon sizes are 359/552/616 bp for Hb Lepore Hollandia, 552/616 bp for Hb Lepore Baltimore, and 616 bp for Hb Lepore Boston - Washington. In this representative sample, positivity for the Hb Lepore Boston - Washington variant (616 bp) was identified, while the Hb Lepore Hollandia and Hb Lepore Baltimore lanes were negative. The wild-type control band (439 bp) is also shown.
